# Jian-Pi-Yi-Shen Formula Alleviates Chronic Kidney Disease in Two Rat Models by Modulating QPRT/NAD^+^/SIRT3/Mitochondrial Dynamics Pathway

**DOI:** 10.1155/2021/6625345

**Published:** 2021-12-13

**Authors:** Xinhui Liu, Siqi Liu, Bing Zhang, Denggui Luo, Shiying Huang, Fochang Wang, Lin Zheng, Jiandong Lu, Jianping Chen, Shunmin Li

**Affiliations:** ^1^Department of Nephrology, Shenzhen Traditional Chinese Medicine Hospital, Guangzhou University of Chinese Medicine, Shenzhen, Guangdong, China; ^2^Shenzhen Key Laboratory of Hospital Chinese Medicine Preparation, Shenzhen Traditional Chinese Medicine Hospital, Guangzhou University of Chinese Medicine, Shenzhen, Guangdong, China

## Abstract

**Objective:**

Jian-Pi-Yi-Shen formula (JPYSF) is a traditional Chinese herbal decoction and has been used for treating chronic kidney disease (CKD) in clinics for decades. However, the potential mechanisms have not been fully elucidated. This study was designed to test the efficacy of JPYSF in treating CKD and explore the underlying mechanism.

**Methods:**

Two CKD rat models were established by 5/6 nephrectomy (5/6 Nx) and feeding with adenine-containing feed, respectively. The intervention dose of JPYSF was 10.89 g/kg/d by gastric irrigation. Renal function was assessed by serum creatinine (Scr) and blood urea nitrogen (BUN). Periodic acid-Schiff (PAS) and Masson's trichrome staining were used to evaluate renal histopathological changes. The levels of nicotinamide adenine dinucleotide (NAD^+^) were measured by using the enzyme-linked immunosorbent assay kit. The proteins expressions of renal fibrosis, quinolinate phosphoribosyltransferase (QPRT), sirtuin 3 (SIRT3), and mitochondrial dynamics were determined and quantified by Western blot analysis.

**Results:**

The results show that administration of JPYSF significantly lowered Scr and BUN levels, improved renal tubular atrophy and interstitial fibrosis, and decreased renal extracellular matrix deposition in two CKD rat models. In addition, CKD rats exhibited suppressed QPRT/NAD^+^/SIRT3 signal, increased mitochondrial fission, and decreased mitochondrial fusion. JPYSF treatment promoted QPRT/NAD^+^/SIRT3 signal and restored mitochondrial fission/fusion balance.

**Conclusion:**

In conclusion, administration of JPYSF effectively alleviated CKD progression in two rat models, which may be related with regulation of the QPRT/NAD^+^/SIRT3/mitochondrial dynamics pathway.

## 1. Introduction

The new data analysis suggested that in 2017, the global prevalence of chronic kidney disease (CKD) was 9.1% (697.5 million cases); CKD resulted in 1.2 million deaths and was the 12th leading cause of death worldwide [1, 2]. Therefore, CKD is a global public health problem, and seeking effective treatments is urgent. Accumulating evidence indicates that traditional Chinese medicine (TCM) is effective in treating CKD, whether in CKD patients [3–5] or animal models [6–8]. Jian-Pi-Yi-Shen formula (JPYSF) has been used for treating CKD in clinics for decades. Our previous studies have found that JPYSF could protect the kidney from 5/6 nephrectomy-induced CKD by regulating mitochondrial dynamics [9]; however, the underlying mechanism is unclear.

Mitochondria are dynamic organelles constantly undergoing fission and fusion events. Mitochondrial fusion is regulated by mitofusin (MFN) and optic atrophy 1 (OPA-1). The main regulator of mitochondrial fission is dynamin-related protein 1 (Drp-1) [10]. Sirtuin 3 (SIRT3) is a nicotinamide adenine dinucleotide (NAD^+^)-dependent lysine deacetylase and is mainly located in the mitochondrial matrix and inner membrane [11]. Recent studies found that SIRT3 could inhibit mitochondrial fission and promote fusion in the treatment of acute kidney injury (AKI) [12], vitiligo [13], and Huntington's disease [14]. However, the role of SIRT3 in regulating mitochondrial dynamics in the CKD model remains unclear. Given that SIRT3 is regulated by levels of NAD^+^, it is necessary to further test NAD^+^ status and upstream signal. Recently, de novo NAD ^+^ synthesis is considered to be important for health [15, 16]. Poyan Mehr et al. showed that a bottleneck enzyme in de novo NAD ^+^ biosynthesis, quinolinate phosphoribosyltransferase (QPRT), defended renal NAD^+^ and mediated resistance to AKI [17]. We asked the question of whether the QPRT/NAD^+^/SIRT3/mitochondrial dynamics pathway was impaired in CKD and could be regulated by JPYSF treatment. In the present study, we established two CKD rat models by 5/6 nephrectomy (5/6 Nx) and feeding with adenine-containing feed, respectively. The efficacy of JPYSF was first determined, and QPRT/NAD^+^/SIRT3/mitochondrial dynamics pathway was subsequently measured.

## 2. Materials and Methods

### 2.1. Preparation of JPYSF Extract

JPYSF is composed of eight traditional Chinese herbal medicines, including *Astragalus mongholicus* Bunge (Fabaceae), *Atractylodes macrocephala* Koidz. (Asteraceae), *Dioscorea oppositifolia* L. (Dioscoreaceae), *Cistanche deserticola* Ma (Orobanchaceae), *Wurfbainia vera* (Blackw.) Skornick. and A. D. Poulsen (Zingiberaceae), *Salvia miltiorrhiza* Bunge (Lamiaceae), *Rheum palmatum* L. (Polygonaceae), and *Glycyrrhiza uralensis* Fisch. ex DC. (Fabaceae). The plant names have been validated with https://mpns.science.kew.org/mpns-portal/. All raw herbs were weighed and boiled twice in 8x ddH_2_O (w/v) for 1 h per time. Following subsequent centrifugation, the supernatant was dried by a freeze dryer and stored at −80°C. The chemical profile and quality control of JPYSF extract have been well described by using ultraperformance liquid chromatography with quadrupole time-of-flight tandem mass spectrometry (UPLC-Q-TOF-MS/MS) in our previous study [18]. Briefly, a total of 71 compounds were identified from JPYSF extract, including saponins, flavonoids, sesquiterpenoids, coumarins, phenylpropanoids, anthranones, anthraquinones, tannins, phenolic acids, and others. Twelve main constituents were chosen as chemical markers to evaluate the quality of JPYSF extract. The minimum quantities (in *μ*g/g) of these 12 constituents present in JPYSF extract dry powder are 1361.01 for rhein, 519.60 for salvianolic acid A, 251.73 for liquiritin, 98.03 for acteoside, 76.06 for calycosin 7-*O*-glucoside, 61.64 for rosmarinic acid, 41.39 for formononetin, 25.03 for calycosin, 13.05 for astragaloside IV, 8.95 for atractylenolide I, 3.45 for dioscin, and 0.21 for tanshinone IIA. This standard guarantees the quality of JPYSF extract used in our study.

### 2.2. Animals

**Figure 1:**
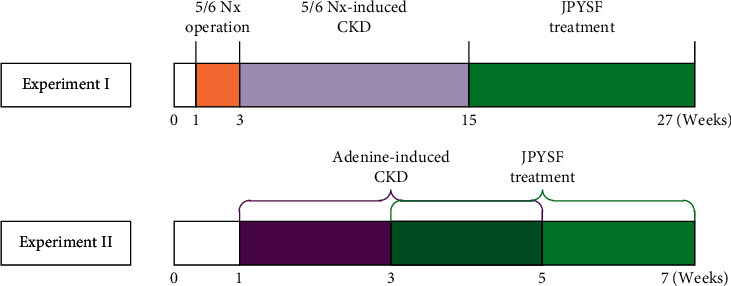
Animal experimental scheme.

All animal experiments were conducted with protocols approved by the Experimental Animal Ethics Committee of Guangzhou University of Chinese Medicine (No. 20190226020). Healthy male Sprague-Dawley (SD) rats weighted 150–180 g were purchased from Guangdong Medical Laboratory Animal Center (Foshan, China). After one week of acclimatization, all rats were randomly assigned to two independent experimental designs. In experiment I, the rats were randomly divided into the following groups (*n* = 6 rats per group): (1) sham group; (2) 5/6 nephrectomy-induced CKD group (5/6 Nx-CKD); (3) JPYSF treatment group (CKD + JPYSF). The 5/6 Nx operation was performed in accordance with our previous publication [9]. The sham group only exposed the kidney without destroying the kidney tissue. After 12 weeks of 5/6 Nx operation, JPYSF extract was administered to CKD rats at the dose of 10.89 g/kg/d for 12 weeks by gastric irrigation. In experiment II, the rats were randomly divided into the following groups (*n* = 6 rats per group): (1) control group; (2) adenine-induced CKD group (adenine-CKD); (3) JPYSF treatment group (CKD + JPYSF). Adenine-induced CKD was performed by feeding adenine (Sigma-Aldrich, St Louis, MO, USA) in feed at a concentration of 0.75% *w/w* for 4 weeks [8, 19]. Rats in the CKD + JPYSF group began to receive JPYSF treatment (10.89 g/kg/d, orally) from the third week for 4 weeks. At the end of experiments, all rats were anesthetized, and blood samples were obtained from abdominal aorta. The rats were euthanized by cervical dislocation without regaining consciousness. Kidneys were rapidly harvested and processed for further analysis. The animal experiment design is shown in Figure 1.

### 2.3. Serum Biochemistry

Serum samples were obtained by centrifuging blood for 10 min at 2,000 rpm at 4°C. The levels of serum creatinine (Scr) and blood urea nitrogen (BUN) were measured by a creatinine serum detection kit and a BUN detection kit (StressMarq Biosciences, British Columbia, Canada), respectively, following the manufacturer's instructions.

### 2.4. Histological Analysis

The paraffin-embedded kidney sections were performed periodic acid-Schiff (PAS) staining and Masson's trichrome staining to evaluate renal pathological injury and tubulointerstitial fibrosis. All images were captured by using the Nikon microscope and NIS-Elements BR software version 4.10.00 (Nikon, Japan).

### 2.5. Western Blotting

The kidney cortexes were homogenized in RIPA buffer (Cell Signaling Technology, Beverly, MA, USA) containing a protease inhibitor cocktail. Equal amounts of kidney cortex lysates were loaded on and electrophoresed through 7% or 10% SDS-PAGE gels and were then transferred to nitrocellulose membranes (Millipore, Billerica, MA, USA). After blocking with 5% skim milk, the membranes were incubated with primary antibodies against fibronectin (FN, 1 : 250), type IV collagen (Col-IV, 1 : 250) (Abcam, Cambridge, MA, USA), *α*-smooth muscle actin (*α*-SMA, 1 : 1000), QPRT (1 : 500), *β*-actin (1 : 5000) (Sigma-Aldrich, St Louis, MO, USA), sirtuin 3 (SIRT3, 1 : 1000) (Proteintech, Wuhan, China), dynamin-related protein 1 (Drp-1, 1 : 1000) (Cell Signaling Technology, Beverly, MA, USA), and optic atrophy 1 (OPA-1, 1 : 2000) (BD Biosciences, San Jose, CA, USA) at 4°C overnight. After incubation with horseradish peroxidase (HRP)-conjugated secondary antibodies (1 : 2000) (Life Technologies, Carlsbad, CA, USA) and Immobilon Western Chemiluminescent HRP Substrate (Millipore, Billerica, MA, USA), the blots were visualized using the ChemiDoc MP Imaging System (BioRad Laboratories, Hercules, CA, USA). Bands were quantified by densitometry using Image Lab software version 5.1 (BioRad Laboratories, Hercules, CA, USA).

### 2.6. Measurement of NAD^+^ and QA

The content of NAD^+^ and quinolinic acid (QA) in kidney tissue was examined using a rat NAD^+^ ELISA kit and a rat QA ELISA kit (Jonln, Shanghai, China), respectively, following the manufacturer's protocol. In brief, the kidney cortex lysate was incubated with HRP-labeled detection antibody for 1 h at 37°C. After washing 5 times with washing buffer, 50 *μ*L each of substrate A and B was added to each well and coincubated at 37°C for 15 min in the dark. Optical density (OD) values were measured using the BioTek microplate reader (BioTek Winooski, Vermont, USA) at 450 nm wavelength. The protein concentration of kidney cortex lysate was measured by the Bradford method. The values of NAD^+^ and QA in the kidney were normalized to the protein concentrations.

### 2.7. Statistical Analysis

Data are expressed as mean ± standard error of the mean (SEM). Data analysis was performed using GraphPad Prism 7.04 software (La Jolla, CA, USA). Comparisons were made using one-way ANOVA with Tukey's post hoc test. Statistical significance was defined as *P* < 0.05.

## 3. Results

### 3.1. JPYSF Improved Renal Function in CKD Rats

**Figure 2:**
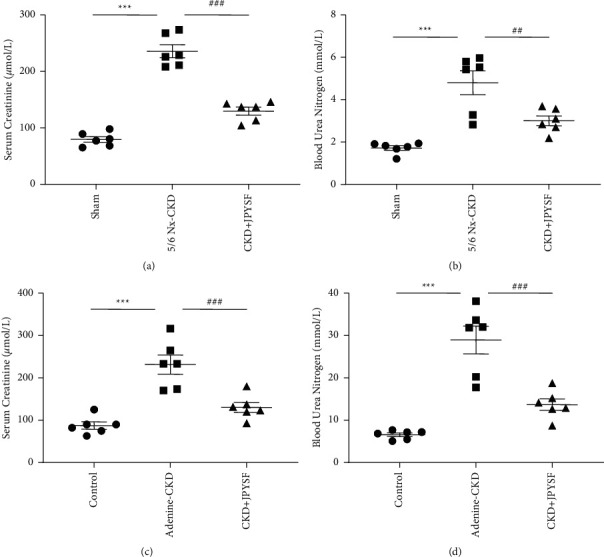
JPYSF improved kidney function in CKD rats. (a) The level of serum creatinine in each group in the setting of 5/6 Nx-induced CKD. (b) The level of blood urea nitrogen in each group in the setting of 5/6 Nx-induced CKD. (c) The level of serum creatinine in each group in the setting of adenine-induced CKD. (d) The level of blood urea nitrogen in each group in the setting of adenine-induced CKD. Data are presented as the means ± SEM, *n* = 6 rats per group (^*∗∗∗*^*P* < 0.001 compared with the sham or the control group; ^##^*P* < 0.01 compared with the 5/6 Nx-CKD group; ^###^*P* < 0.001 compared with the 5/6 Nx-CKD or the adenine-CKD group).

Scr and BUN are the most commonly used clinical indicators to monitor renal function. The levels of Scr and BUN were significantly increased in both 5/6 Nx-induced CKD and adenine-induced CKD rats. After JPYSF treatment, Scr and BUN levels were dramatically reduced compared with the CKD group (*P* < 0.01) (Figure 2).

### 3.2. JPYSF Attenuated Renal Tubulointerstitial Injury in CKD Rats

**Figure 3:**
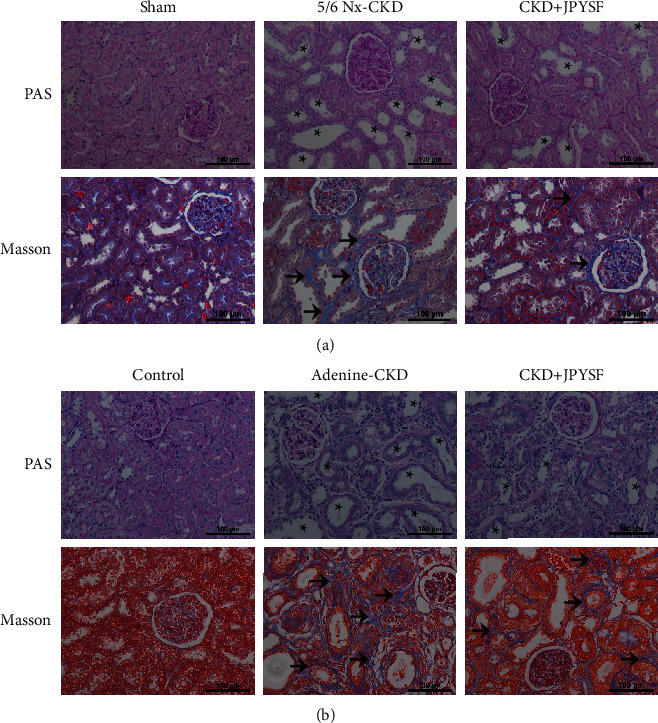
JPYSF ameliorated renal injury in CKD rats. (a) PAS and Masson staining in the setting of 5/6 Nx-induced CKD. (b) PAS and Masson staining in the setting of adenine-induced CKD. Asterisk indicates tubular expansion; arrow indicates collagen fibril. All images are shown at identical magnification, ×200, scale bar = 100 *μ*m.

In PAS staining, massive tubular epithelial cells atrophy, partial renal tubular expansion, and loose tissue arrangement were observed in CKD rats, which could be improved by JPYSF treatment. Masson staining displayed that both 5/6 Nx and adenine lead to an obvious accumulation of collagen fibrils (blue staining) in the renal interstitium. The fibrotic injury in CKD rats was markedly attenuated after JPYSF treatment (Figure 3).

### 3.3. JPYSF Decreased Renal Extracellular Matrix Deposition in CKD Rats

**Figure 4:**
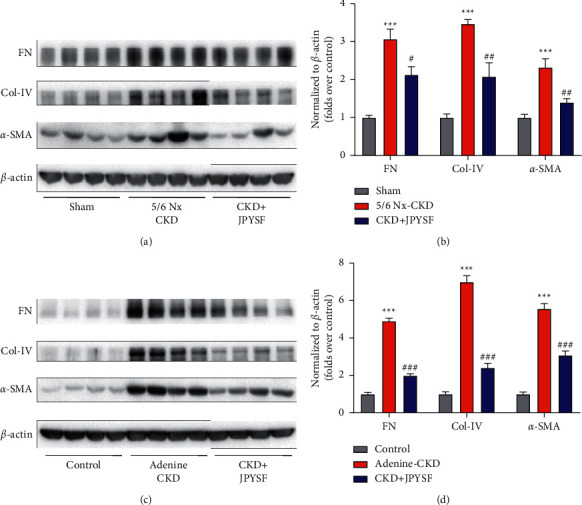
JPYSF suppressed the expression of fibrotic markers in the kidney of CKD rats. Representative Western blot images (a) and densitometric analyses (b) of FN, Col-IV, and *α*-SMA protein expression in the setting of 5/6 Nx-induced CKD. Representative Western blot images (c) and densitometric analyses (d) of FN, Col-IV, and *α*-SMA protein expression in the setting of adenine-induced CKD. All proteins expression was normalized to *β*-actin content. Data are presented as the means ± SEM, *n* = 6 rats per group (^*∗∗∗*^*P* < 0.001 compared with the sham or the control group; ^#^*P* < 0.05 compared with the 5/6 Nx-CKD group; ^##^*P* < 0.01 compared with the 5/6 Nx-CKD group; ^###^*P* < 0.001 compared with the adenine-CKD group).

Renal fibrosis is a hallmark of CKD and is characterized by extracellular matrix deposition. We assessed FN, Col-IV, and *α*-SMA expression by Western blot analysis in the kidney of CKD rats with or without JPYSF treatment. CKD rats had significantly increased FN, Col-IV, and *α*-SMA expression compared with the sham or control group. However, JPYSF treatment markedly decreased these proteins expression in the kidney of CKD rats (Figure 4).

### 3.4. JPYSF Promoted QPRT/NAD^+^/SIRT3 Signal in CKD Rats

**Figure 5:**
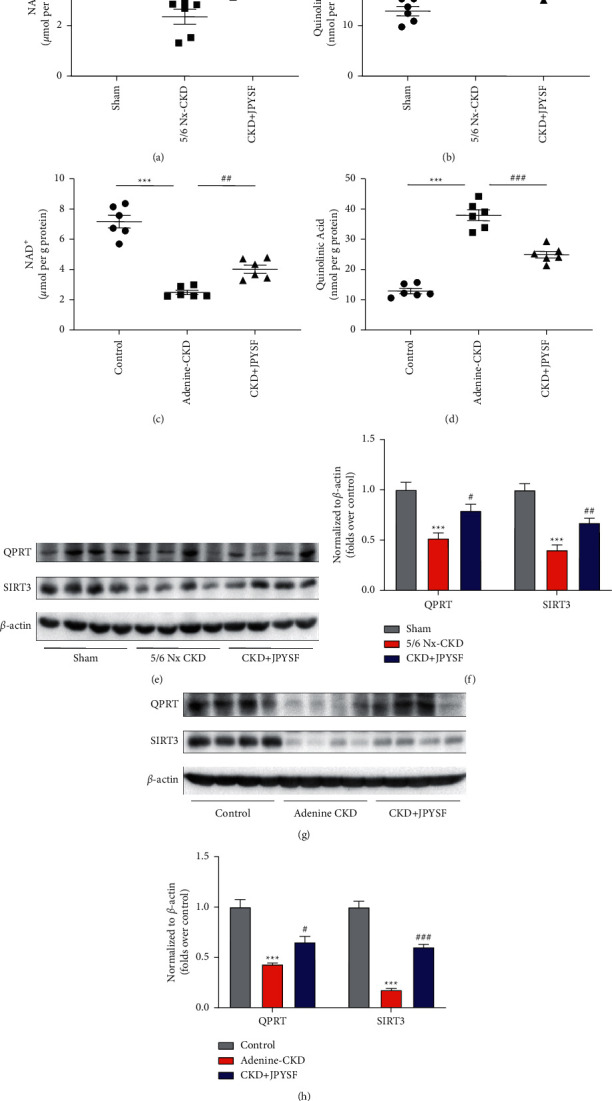
Effect of JPYSF on the QPRT/NAD^+^/SIRT3 pathway in the kidney of CKD rats. The levels of NAD^+^ (a) and quinolinic acid (b) in each group in the setting of 5/6 Nx-induced CKD. The levels of NAD^+^ (c) and quinolinic acid (d) in each group in the setting of adenine-induced CKD. Representative Western blot images (e) and densitometric analyses (f) of QPRT and SIRT3 protein expression in the setting of 5/6-Nx induced CKD. Representative Western blot images (g) and densitometric analyses (h) of QPRT and SIRT3 protein expression in the setting of adenine-induced CKD. All proteins expression was normalized to *β*-actin content. Data are presented as the means ± SEM, *n* = 6 rats per group (^*∗∗∗*^*P* < 0.001 compared with the sham or the control group; ^#^*P* < 0.05 compared with the 5/6 Nx-CKD or the adenine-CKD group; ^##^*P* < 0.01 compared with the 5/6 Nx-CKD or the adenine-CKD group; ^###^*P* < 0.001 compared with the adenine-CKD group).

The level of NAD^+^ in the kidney of CKD rat was significantly lower compared with normal rat (Figures 5(a) and 5(c)). On the contrary, the content of QA, NAD ^+^ precursor in de novo biosynthesis, was elevated in the kidney of CKD rat (Figures 5(b) and 5(d)). Not surprisingly, the expression of the enzyme QPRT that catalyzes QA was downregulated in CKD rats. In addition, NAD^+^-dependent deacetylase SIRT3 was also downregulated in CKD rats (Figures 5(e)–5(h)). Administration of JPYSF markedly increased QPRT expression, lowered QA content, increased NAD^+^ level, and upregulated SIRT3 expression in CKD rats. These results indicated that QPRT/NAD^+^/SIRT3 signal was suppressed in the kidney of CKD rats and could be promoted by JPYSF treatment.

### 3.5. JPYSF Modulated Mitochondrial Dynamics in CKD Rats

**Figure 6:**
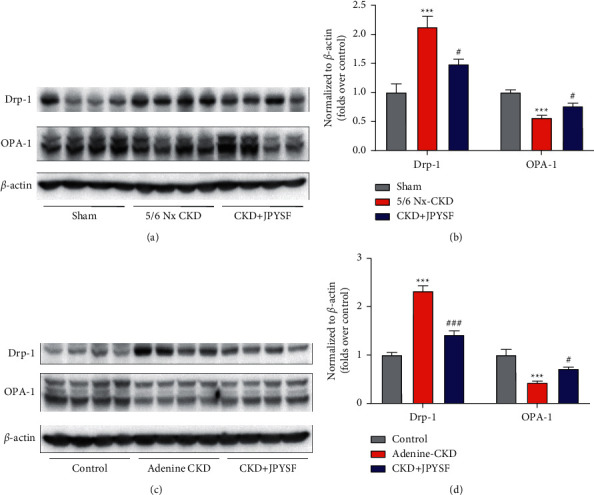
JPYSF modulated mitochondrial dynamics in CKD rats. Representative Western blot images (a) and densitometric analyses (b) of Drp-1 and OPA-1 protein expression in the setting of 5/6 Nx-induced CKD. Representative Western blot images (c) and densitometric analyses (d) of Drp-1 and OPA-1 protein expression in the setting of adenine-induced CKD. All proteins expression was normalized to *β*-actin content. Data are presented as the means ± SEM, *n* = 6 rats per group (^*∗∗∗*^*P* < 0.001 compared with the sham or the control group; ^#^*P* < 0.05 compared with the 5/6 Nx-CKD or the adenine-CKD group; ^###^*P* < 0.001 compared with the adenine-CKD group).

Mitochondrial dynamics was governed by the balance of mitochondrial fission and fusion. Drp-1 and OPA-1 are the key regulators of mitochondrial fission and fusion, respectively. Our data found that Drp-1 was upregulated and OPA-1 was downregulated in CKD rats. JPYSF treatment could reverse the expression pattern of Drp-1 and OPA-1 (Figure 6). This result suggested that JPYSF could modulate mitochondrial dynamics in CKD rats.

## 4. Discussion

In the present study, two CKD rat models were successfully established by 5/6 Nx and feeding with adenine-containing feed. Administration of JPYSF improved renal function and pathological injury and decreased renal extracellular matrix deposition in CKD rats. In addition, CKD rats exhibited suppressed QPRT/NAD^+^/SIRT3 signal, increased mitochondrial fission, and decreased mitochondrial fusion. JPYSF treatment promoted QPRT/NAD^+^/SIRT3 signal and restored mitochondrial fission/fusion balance.

TCM believes that the basic pathogenesis of CKD is deficiency of spleen and kidney Qi, dampness, blood stasis, and turbidity. JPYSF was founded based on this theory and has the effect of invigorating the spleen and kidney, promoting blood circulation, and removing turbidity. In order to obtain more solid evidence, we used two rat models to study the efficacy and mechanism of JPYSF in the treatment of CKD. Both 5/6 Nx and adenine feed can induce CKD phenotype [20–22]; the difference is that 5/6 Nx requires surgery and a longer experimental period. In this study, these two CKD models were successfully established, and JPYSF could effectively improve CKD (Figures 2 and 3), which laid the foundation for mechanism research.

The unbalanced mitochondrial fission/fusion is consistent with our previous results [8, 9], but the regulatory mechanism is still unclear. Sirtuins are an evolutionarily conserved family comprising of seven members (SIRT1–SIRT7) in mammals, among which three (SIRT3, SIRT4, and SIRT5) are exclusively localized to mitochondria [23]. Striking mitochondrial protein hyperacetylation was detected in SIRT3-deficient mice but not SIRT4 and SIRT5-deficient mice, suggesting that SIRT3 is a major mitochondrial deacetylase [11]. SIRT3 has been documented as an important molecule in regulating mitochondrial function with the implications in metabolic enzyme activity, oxidative phosphorylation efficacy, and antioxidant machinery [24–26]. Emerging evidence indicates that SIRT3 is critical in sustaining mitochondrial dynamics. In the cisplatin-induced AKI mice model, renal SIRT3 was downregulated leading to increased expression of Drp-1, accompanied by a decrease in OPA-1, thus carrying mitochondria dynamics toward fission and fragmentation. Upregulation of SIRT3 resulted in mitochondrial fusion, limiting segregation and protection against cisplatin-induced AKI [12]. Specific activation of SIRT3 by honokiol was capable to protect vitiligo melanocytes against oxidative stress via the regulation of OPA-1-dependent mitochondrial dynamics [13]. SIRT3 could alleviate renal ischemia-reperfusion injury through enhancing mitochondrial fusion and activating the ERK-OPA-1 signaling pathway [27]. Recently, SIRT3 was found to confer neuroprotection in Huntington's disease by regulation of mitochondrial dynamics [14]. Consistent with previous studies, our data show that SIRT3 was downregulated, accompanied by an increase in Drp-1 and decrease in OPA-1 in CKD rat models. However, the specific mechanism by which SIRT3 regulates mitochondrial dynamics needs further study.

As a NAD^+^-dependent deacetylase, the function of SIRT3 is regulated by levels of NAD^+^. Intracellular NAD^+^ can be produced through either de novo synthesis from tryptophan or via salvage pathways from precursor molecules such as nicotinamide, nicotinic acid, or nicotinamide riboside [28]. Klimova et al. reported that NAD ^+^ precursor, nicotinamide mononucleotide, altered mitochondrial dynamics in the brain by reducing fragmentation of neuronal mitochondria by the SIRT3-dependent mechanism [29]. In recent years, the critical role of de novo NAD ^+^ synthesis in health and disease has been gradually revealed [15–17]. A well-designed study found that impairment of NAD^+^ synthesis is a hallmark of AKI and CKD. However, nicotinamide riboside supplementation aimed to boost NAD^+^ production via salvage pathways was beneficial in ischemic AKI but not in CKD models [30]. This study suggested that impaired de novo NAD ^+^ synthesis played more important role in CKD. In de novo NAD ^+^ synthesis, tryptophan and other upstream metabolites have multiple metabolic fates, making QA the first fully committed NAD ^+^ precursor in de novo biosynthesis. QPRT is the enzyme that connects the initial steps of opening QA's pyrrole ring to the final steps of NAD^+^ biosynthesis [31]. Similar with a previous study in AKI, our data also found low expression of renal QPRT, QA accumulation, and low NAD^+^ levels in CKD models, which suggested that de novo NAD ^+^ synthesis was impaired in CKD and may represent therapeutic target for CKD treatment.

## 5. Conclusions

In conclusion, administration of JPYSF effectively alleviated CKD progression in two rat models, which may be related with regulation of the QPRT/NAD^+^/SIRT3/mitochondrial dynamics pathway.

## Data Availability

The data used to support the findings of this study are available from the corresponding author upon request.

## Abbreviations

5/6 Nx: 5/6 Nephrectomy
*α*-SMA: *α*-Smooth muscle actin
AKI: Acute kidney injury
BUN: Blood urea nitrogen
CKD: Chronic kidney disease
Col-IV: Type IV collagen
Drp-1: Dynamin-related protein 1
FN: Fibronectin
JPYSF: Jian-Pi-Yi-Shen formula
MFN: Mitofusin
NAD: Nicotinamide adenine dinucleotide
OPA-1: Optic atrophy 1
PAS: Periodic acid-Schiff
QA: Quinolinic acid
QPRT: Quinolinate phosphoribosyltransferase
SIRT3: Sirtuin 3
Scr: Serum creatinine
TCM: Traditional Chinese medicine.
